# Cardiovascular sequalae in uncomplicated COVID-19 survivors

**DOI:** 10.1371/journal.pone.0246732

**Published:** 2021-02-11

**Authors:** Mi Zhou, Chun-Ka Wong, Ka-Chun Un, Yuk-Ming Lau, Jeffrey Chun-Yin Lee, Frankie Chor-Cheung Tam, Yee-Man Lau, Wing-Hon Lai, Anthony Raymond Tam, Yat-Yin Lam, Polly Pang, Teresa Tong, Milky Tang, Hung-Fat Tse, Deborah Ho, Ming-Yen Ng, Esther W. Chan, Ian C. K. Wong, Chu-Pak Lau, Ivan Fan-Ngai Hung, Chung-Wah Siu

**Affiliations:** 1 Cardiology Division, Department of Medicine, the University of Hong Kong, Hong Kong SAR, China; 2 Cardiac Medical Unit, the Grantham Hospital, Hong Kong SAR, Hong Kong; 3 Infectious Disease Division, Department of Medicine, the University of Hong Kong, Hong Kong SAR, China; 4 Asian Heart Center, Hong Kong SAR, China; 5 Department of Diagnostic Radiology, the University of Hong Kong, Hong Kong SAR, China; 6 Department of Pharmacology and Pharmacy, Centre for Safe Medication Practice and Research, the University of Hong Kong, Hong Kong SAR, China; Scuola Superiore Sant’Anna, ITALY

## Abstract

**Background:**

A high proportion of COVID-19 patients were reported to have cardiac involvements. Data pertaining to cardiac sequalae is of urgent importance to define subsequent cardiac surveillance.

**Methods:**

We performed a systematic cardiac screening for 97 consecutive COVID-19 survivors including electrocardiogram (ECG), echocardiography, serum troponin and NT-proBNP assay 1–4 weeks after hospital discharge. Treadmill exercise test and cardiac magnetic resonance imaging (CMR) were performed according to initial screening results.

**Results:**

The mean age was 46.5 ± 18.6 years; 53.6% were men. All were classified with non-severe disease without overt cardiac manifestations and did not require intensive care. Median hospitalization stay was 17 days and median duration from discharge to screening was 11 days. Cardiac abnormalities were detected in 42.3% including sinus bradycardia (29.9%), newly detected T-wave abnormality (8.2%), elevated troponin level (6.2%), newly detected atrial fibrillation (1.0%), and newly detected left ventricular systolic dysfunction with elevated NT-proBNP level (1.0%). Significant sinus bradycardia with heart rate below 50 bpm was detected in 7.2% COVID-19 survivors, which appeared to be self-limiting and recovered over time. For COVID-19 survivors with persistent elevation of troponin level after discharge or newly detected T wave abnormality, echocardiography and CMR did not reveal any evidence of infarct, myocarditis, or left ventricular systolic dysfunction.

**Conclusion:**

Cardiac abnormality is common amongst COVID-survivors with mild disease, which is mostly self-limiting. Nonetheless, cardiac surveillance in form of ECG and/or serum biomarkers may be advisable to detect more severe cardiac involvement including atrial fibrillation and left ventricular dysfunction.

## Introduction

Coronavirus Disease 2019 (COVID-19) due to severe acute respiratory syndrome coronavirus 2 (SARS-CoV-2) emerged in December 2019, and in a mere few months has resulted in a pandemic of viral pneumonia. As of November 12, 2020, over 52 million people were infected and more than 241,809 deaths were reported globally [1]. While COVID-19 primarily affects the respiratory system, it also affects multiple organ systems, particularly the cardiovascular system [2–6]. Individuals with pre-existing cardiovascular comorbidities were reported to have poorer outcomes [7–9], and SARS-CoV-2 infection may manifest as acute myocarditis in otherwise healthy individuals [10]. In the early case series from China, up to 27.8% COVID19 patients had an elevated troponin level above the 99^th^ percentile of upper reference limit indicating acute myocardial damage [11, 12]. This is nearly 10-folded higher than that of influenza (2.9%) [13]. In contrast, the majority of COVID-19 patients including those with biochemical evidence of acute myocardial injury have a relatively mild disease course and recover uneventfully without overt cardiac complications. It is unknown whether there is any subclinical and/or latent cardiac damage in COVID-19 survivors without overt cardiac manifestations, which may affect the long-term outcomes. As the pandemic progresses into its deceleration phase, it is of urgent importance to define whether cardiac surveillance may be advisable in COVID-19 survivors. In the present study, we prospectively and systematically screened COVID-19 survivors to detect cardiac abnormalities using serum biomarkers, electrocardiogram (ECG), treadmill exercise test, echocardiography and cardiac magnetic resonance (CMR) imaging. Our results may inform early planning for cardiac surveillance after acute phase of COVID-19.

## Methods

The study protocol conforms to the ethical guidelines of the Declaration of Helsinki. The study protocol was approved by the institutional review board of the University of Hong Kong/Hospital Authority Hong Kong West Cluster.

### Study design and patients

This prospective screening study was conducted at Queen Mary Hospital (QMH), a teaching hospital of The University of Hong Kong. QMH is the only hospital to provide emergency medical service in the Hong Kong West Cluster that serves a population of 530,000 [14]. Patients with PCR-confirmed COVID-19 1–4 weeks after hospital discharge were referred to the Infectious Diseases clinic, QMH and were invited to participate to the study. Patients were excluded if they were below 18 years of age, were classified as severe community acquired pneumonia according to the Clinical Practicing Guideline from the American Thoracic Society [15], required invasive or non-invasive ventilatory support or admitted to intensive care unit during the COVID-19 hospitalization, and/or had history of heart failure. Informed consent was obtained from all recruited participants. In Hong Kong, all COVID-19 patients were managed in negatively pressured isolation wards in public hospitals under the Hospital Authority, Hong Kong. COVID-19 patients were discharged after fulfilling the current discharge criteria including (1) Clinical criteria, that is when the clinical conditions of the patients improve and afebrile for at least 24 hours and (2) Laboratory evidence of SARS-CoV-2 clearance as evidenced by negative RT-PCR tests for nasopharyngeal swab with sampling interval ≥ 24 hours.

### Serum cardiac biomarkers

COVID-19 survivors attended the first follow-up visit at the Infectious Disease clinic 7–28 days after the index hospital discharge date. Demographic data, detailed medical histories, and clinical data pertaining to the COVID-19 hospitalization were retrieved from the territory-wide information network of all public hospitals in Hong Kong. Blood was drawn for serum creatinine, troponin T, N-terminal-B-type natriuretic peptide (NT-proBNP) assayed (Roche Diagnostics, GmbH). Serum troponin level above 99^th^ percentile of the upper reference limit was considered elevated. Serum NT-proBNP level was considered elevated using to the age-specific diagnostic threshold for heart failure.

### ECG and echocardiography

ECG and echocardiography were performed for all study participants on the same day. A standard 12-lead ECG was recorded with the patient at rest in a supine position at paper speed of 25 mm/s and calibration of 1 mV/10 mm. Sinus bradycardia and significant sinus bradycardia are defined as heart rate below 60 bpm and below 50 bpm respectively. All ECGs were independently evaluated by two cardiologists for the presence of bundle branch block, and inverted T waves (negative T wave >0.1 mV in leads other than aVR, aVL, III, and V1). Echocardiograms were obtained using Vivid i ultrasound systems (GE Medical Systems, Israel Ltd, Tirat Carmel, Israel), including two-dimensional, M-mode, and color Doppler images according to the guidelines of American Society of Echocardiography [16, 17]. The average of three cardiac cycles was used for measurements of cardiac dimensions, and left ventricular ejection fraction (LVEF).

### Cardiac magnetic resonance imaging

Contrast enhanced CMR was performed for COVID-19 survivors with persistent elevation of troponin level or newly detected T-wave abnormality 1–2 weeks after the initial cardiac assessment. Cine, T2 short tau inversion recovery (T2 STIR) and late gadolinium enhancement (LGE) images were performed. Cine images were used to determine LV volumes, LVEF and myocardial mass. T2 STIR and LGE images were used to determine the presence of myocarditis or infarcts.

### Treadmill exercise test

Treadmill exercise tests were performed for COVID-19 survivors with significant sinus bradycardia (heart rate < 50 bpm) 1–2 weeks after the initial cardiac assessment according to the chronotropic assessment exercise protocol [18]. Chronotropic incompetence was defined as failure to achieve 85% of the age-predicted heart rate.

### Statistical analysis

Continuous were expressed as mean ± standard deviation for normally distributed variables and median ± interquartile range (IQR), and discrete variables were expressed in percentages, respectively. Chi-square test or Fisher’s exact test was used to compare categorical variables between groups. Student’s t test was performed to compare continuous variables. The association between baseline variables and newly detected cardiac abnormalities was analysed by bivariate and multivariate correlation. Binary logistic regression model was used to calculate hazard ratios (HRs) of predictive factors and the respective 95% confidence interval (CIs) for the newly detected cardiac abnormalities. A *p*-value <0.05 was considered statistically significant. All statistical analyses were performed using the Statistical Package for the Social Sciences software version 21.0 for Mac (SPSS, Chicago, IL).

## Results

**Fig 1:**
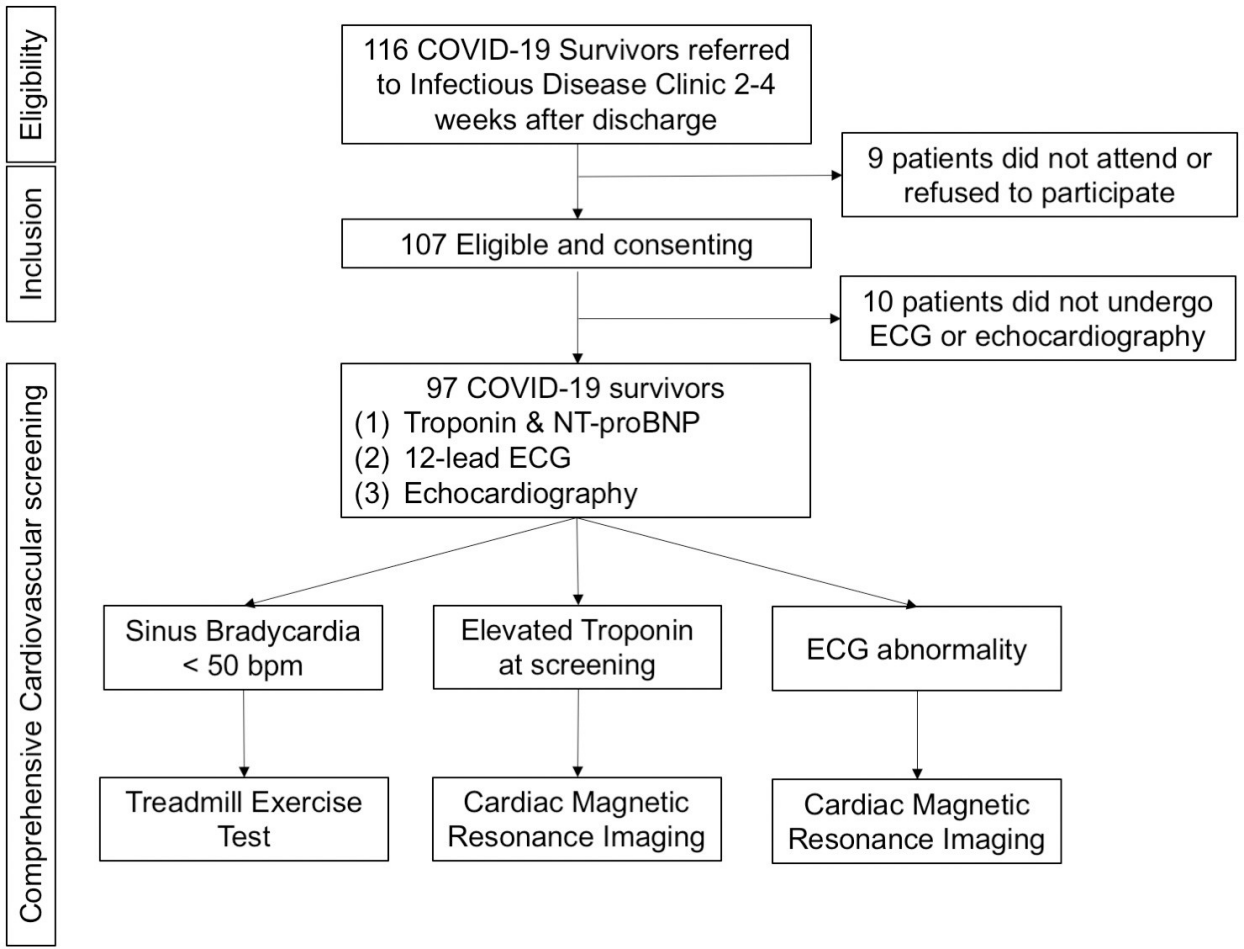
Study population and the screening procedure.

In April 2020, there were 1,036 confirmed COVID-19 cases in Hong Kong, in which 699 cases were discharged from hospital and 4 cases died. Of 695 COVID-19 survivors, 116 (16.3%) were referred to the Infectious Diseases clinic, QMH; 9 patients did not attend the scheduled appointment or refused to participate in the study (Fig 1). In addition, 10 COVID-19 survivors consented for the study but did not undergo ECG and echocardiography. The final analysis included 97 COVID-19 survivors. The mean age was 46.5 ± 18.6 years and 52 (53.6%) were men. Table 1 summarizes the clinical characteristics of the study population. The 22 COVID-19 survivors had history of hypertension (22.1%), diabetes (12, 10.6%), and coronary artery disease (7, 6.2%). On admission, 51 (47.7%) COVID-19 survivors had fever, and 64 (59.8%) had cough. All were classified as non-severe community acquired pneumonia. The mean systolic and diastolic blood pressure were 137.4 ± 18.2 mmHg and 81.6 ± 11.5 mmHg with mean heart rate of 85.9 ± 14.4 bpm. Serum troponin level was measured in 69 out of 97 COVID-19 survivors during the index hospitalization and elevated troponin level was founded in 7 patients (10.1%). No patient had venous thromboembolism. They were discharged from the hospitals after fulfilling the current discharge criteria for COVID-19 patients in Hong Kong with a median hospital stay of 17 days (13–23 days) and referred to the Infectious Diseases clinic for further follow-up.

**Table 1:** Clinical characteristics of the COVID-19 survivors on admission.

	(n = 97)
**Demographics and co-morbidities**	
Age, years,	46.5 ± 18.6; 47 (30–62)
Male, n (%)	52 (53.6)
Hypertension, n (%)	24 (24.7)
Diabetes mellitus, n (%)	11 (11.3)
Chronic kidney disease	1 (1.0)
Coronary artery disease, n (%)	6 (6.2)
Stroke, n (%)	1 (1.0)
History of malignancy, n (%)	2 (2.1)
**Symptoms**	
Fever, n (%)	46 (47.4)
Cough, n (%)	57 (58.8)
Sore throat, n (%)	15 (15.5)
Sputum, n (%)	17 (17.5)
Shortness of breath, n (%)	8 (8.2)
Diarrhea, n (%)	11 (11.3)
Myalgia, n (%)	14 (14.4)
Loss of taste and/or smell, n (%)	9 (9.3)
**Radiographic findings**	
Ground-glass opacity	13 (13.4)
Local patchy shadowing	13 (13.4)
Bilateral patchy shadowing	21 (22.6)
Interstitial abnormalities	0 (0.0)
**Laboratory findings**	
White cell counts, 1x10^9^/ml	6.7 ± 2.5, 6.4 (5.2–8.0)
Lymphocyte counts, 1x10^9^/ml	1.8 ± 0.6, 1.73 (1.25–2.23)
Platelet counts, 1x10^12^/ml	289 ± 105, 278 (211–334)
Alanine aminotransferase U/L	38.8 ± 33.4, 27 (18–44)
Creatinine, umol/L	77.5 ± 17.6, 76 (65–85)
Elevated troponin during hospitalization, (%)^†^	7 (10.1)

Mean ± SD, and median and interquartile range (IQR) were provided for continuous variables.

^†^Total number of patients with serum troponin assay during COVID-19 hospitalization was 69.

### Cardiac arrhythmias

**Fig 2:**
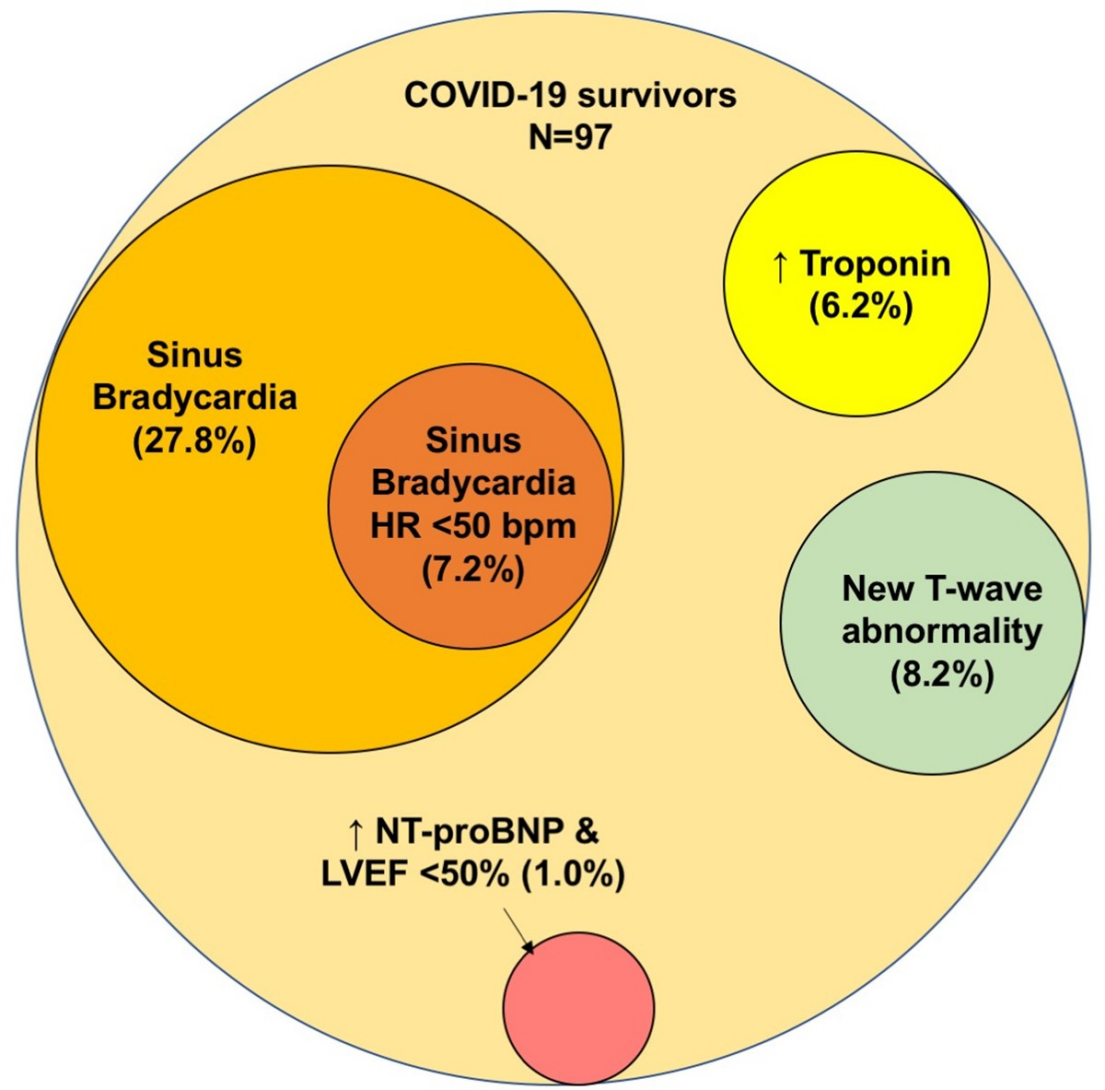
Latent cardiac abnormalities amongst 97 COVID-19 survivors 2–4 weeks after discharge for the index hospitalization for COVID-19.

The median duration from discharge to the cardiac surveillance was 11 days (8–35). Sixty-six (68%) were in normal sinus rhythm, 29 (29.9%) were in sinus bradycardia, and 2 (2.1%) were in atrial fibrillation (AF) (Table 2). One COVID-19 survivor had a newly detected left bundle branch block (Table 3). Of note, 2 COVID-19 survivors found to have sinus bradycardia were on beta-adrenergic blocker for hypertension; 1 of the 2 with AF had documented AF prior to COVID-19 (Table 2). The remaining 27 COVID-19 survivors (27.8%) with sinus bradycardia not related to medication had a mean age of 44.1 ± 14.3 years and 81.5% were men. The mean heart rate was 53.5 ± 5.1 bpm, and 3 had concomitant prolongation of PR-interval >200 msec. Except for the difference in the proportion of men (81.5% vs. 42.9%, *p* = 0.001) among the COVID-19 survivors with or without sinus bradycardia, no other significant differences were found in aged or other comorbidities. Furthermore, 7 COVID-19 survivors (7.2%) had significant sinus bradycardia (heart rate < 50 bpm) (Tables 2 and 3, Fig 2). None had pre-syncope or syncope after discharge. Five underwent treadmill exercise test to evaluate chronotropic response 1–2 weeks later. At the time of the treadmill exercise test, 3 had a resting heart rate above 60 bpm with normal chronotropic response and 2 with sinus bradycardia demonstrated an improvement in resting heart rate which improved to 52 and 58 bpm respectively. Regardless, their chronotropic indexes were only 70% and 75% respectively, indicating chronotropic incompetency.

**Table 2:** Cardiac abnormalities detected during screening.

**Serum Cardiac biomarkers**	N = 97	
Troponin > 99^th^ percentile of ULN	6 (6.2)	
Elevated NT-proBNP > age-specific diagnostic threshold of heart failure	1 (0.9)	
**Electrocardiography**	N = 97	
	Total	Newly detected
Sinus bradycardia (<60 bpm), n (%)^†^	27 (27.8)	27 (27.8)
Sinus bradycardia (<50 bpm), n (%)^†^	7 (7.0)	7 (7.0)
Atrial fibrillation, n (%)	2 (2.1)	1 (1.0)
LBBB, n (%)	1 (1.0)	1 (1.0)
RBBB, n (%)	1 (1.0)	0 (0.0)
T wave inversion, n (%)		
Anterior leads, n (%)	10 (10.3)	8 (8.2)
Lateral leads, n (%)	2 (2.1)	0 (0.0)
Inferior leads, n (%)	2 (2.1)	1 (1.0)
Any leads, n (%)	12 (12.4)	8 (8.2)
**Echocardiographic findings**	n = 97	
Left ventricular dimension, cm	4.4 ± 0.5	
Left ventricular ejection fraction, %	66.2 ± 7.1	
Left ventricular ejection <50%	1 (1.0%)	

^†^Patients on antiarrhythmic drugs were excluded.

Anterior leads inverted T waves: Leads V1 through V3.

Lateral leads inverted T waves: Leads V4 through V6.

Inferior leads inverted T waves: Leads II, III, and aVF.

**Table 3:** Newly detected cardiac abnormalities.

Case	Demographic and Pre-existing comorbidities					Newly detected cardiac abnormalities						
	Age (years)	Sex	HT (+/−)	DM (+/−)	CAD (+/−)	Sinus bradycardia HR<50 bpm (+/−)	AF (+/−)	ST-T abnormality (+/−)	LBBB (+/−)	Elevated troponin (+/−)	Elevated NT-proBNP (+/−)	LVEF<50% (+/−)
**1**	18	F	−	−	−	−	−	Anterior	−	−	−	−
**2**	20	M	−	−	−	+ (46 bpm)	−	−	−	−	−	−
**3**	34	M	−	−	−	+ (44 bpm)	−	−	−	−	−	−
**4**	34	M	−	−	−	+ (49 bpm)	−	−	−	−	−	−
**5**	35	F	+	−	−	−	−	Anterior	−	−	−	−
**6**	38	M	−	−	−	+ (45 bpm)	−	−	−	−	−	−
**7**	48	F	−	−	−	−	−	Anterior	−	−	−	−
**8**	57	F	−	−	−	−	−	Anterior	−	−	−	−
**9**	57	F	−	−	−	−	−	−	+	−	−	−
**10**	59	M	−	−	−	+ (48 bpm)	−	−	−	−	−	−
**11**	61	F	−	−	−	−	−	Anterior + inferior	−	−	−	−
**12**	61	F	−	−	−	−	−	Anterior	−	−	−	−
**13**	64	M	+	+	−	+ (48 bpm)	−	−	−	−	−	−
**14**	66	M	+	+	−	+ (48 bpm)	−	−	−	−	−	−
**15**	68	F	−	−	−	−	−	Anterior	−	−	−	−
**16**	68	M	+	−	+	−	−	Anterior	−	−	−	−
**17**	68	M	+	+	−	−	−	−	−	+ (16 ng/L)	−	−
**18**	69	M	+	−	−	−	+^††^	−	−	−	+	+ (44%)
**19**	69	M	+	+	−	−	−	−	−	+ (21 ng/L)^†^	−	−
**20**	71	M	+	−	+	−	−	−	−	+ (19 ng/L)	−	−
**21**	76	M	+	+	+	−	+	−	−	+ (15 ng/L)	−	−
**22**	84	M	−	−	−	−	−	−	−	+ (16 ng/L)	−	−
**23**	87	M	+	−	−	−	−	−	−	+ (61 ng/L)	−	−

Abbreviations: AF: Atrial fibrillation; CAD: Coronary artery disease; DM: Diabetes mellitus; F: Female; M: Male; HR: Heart rate; HT: Hypertension; LBBB: Left bundle branch block; LVEF: Left ventricular ejection fraction; +/−: Presence/absence.

†Late gadolinium contrast enhancement over anterior wall on cardiac magnetic resonance imaging.

††Known AF.

### High-sensitive troponin level

Six (6.2%) out of 97 COVID-19 survivors were detected to have a troponin level above 99^th^ percentile of upper reference limit at the cardiac surveillance (Table 3). In fact, troponin level was measured in 4 out of these 6 COVID-19 survivors during the COVID-19 hospitalization, which were all elevated. Compared with COVID-19 survivors with normal troponin level, those with an elevated troponin level were older (75.8 ± 8.0 years *vs*. 43.8 ± 17.1 years, *p*<0.001), more likely to have hypertension (83.3% *vs*. 19.8%, *p*<0.001), diabetes (50.0% *vs*. 8.9%, *p* = 0.018), and pre-existing coronary artery disease (33.3% *vs*. 4.0%, *p* = 0.036). Nonetheless, all 6 COVID-19 survivors with elevated troponin level had normal NT-proBNP level and preserved left ventricular systolic with LVEF >50% at the cardiac surveillance. To further evaluate the underlying cause of persistent elevation of troponin level, CMR was performed in 4 of these 6 COVID-19 survivors. For the 2 COVID-19 survivors not undergoing cardiac magnetic resonance imaging, one was implanted with CMR-noncompatible pacemaker and the other refused to participate due to claustrophobia. CMR showed no infarcts in all 4 COVID-19 survivors. Three COVID-19 survivors showed no features of myocarditis. One had a small focus of sub-epicardial LGE in the basal anterolateral wall but no corresponding high T2 STIR changes. Thus, there was no evidence of acute myocarditis although previous or resolved myocarditis could not be ruled out.

### Left ventricular dysfunction and NT-proBNP level

For the 97 COVID-19 survivors undergoing echocardiography, the mean LVEF was 66.4 ± 6.8%. One (1.0%) had impaired left ventricular systolic function with LVEF 44% together with an elevated NT-proBNP level above the age-specific diagnostic threshold of heart failure (Tables 2 and 3, Fig 2).

### Newly detected T-wave inversion

T-wave inversion were detected 12 COVID-19 survivors (10.3%), of which 4 existed in prior ECGs (Tables 2, and 3). New T-wave inversion were found in the anterior leads in all 8 COVID-19 survivors with 1 with co-existing T-wave inversions in inferior leads. The mean age was 52.0 ± 17.5 years. COVID-19 survivors with newly detected T-wave inversion were more likely to be women (87.5% vs. 42.7%, *p* = 0.023), but there was no difference found in age, and other comorbidities amongst those without newly detected T-wave inversion. Serum troponin level was measured in 7 of them during the hospitalization for COVID-19, which were all below the 99^th^ percentile of upper reference limit. Lastly, no elevated troponin/NT-proBNP level, or impaired left ventricular systolic function was found during at the cardiac surveillance. Furthermore, CMR showed no infarcts or myocarditis in all 8 COVID-19 survivors with newly detected T-wave abnormality.

### Factors for newly detected cardiac abnormalities

Table 4 summarizes the factors associated with predictive newly detected cardiac abnormalities among the study cohort. On univariate analysis, increasing age (HR: 1.04, 95% CI: 1.01–1.07, *p* = 0.003*) and pre-existing hypertension (HR: 3.24, 95% CI: 1.12–8.90, *p* = 0.022*) were statistically significantly associated with newly detected cardiac abnormalities, but only increasing age remained associated with newly detected cardiac abnormalities in multivariate analysis.

**Table 4:** Factors for newly detected cardiac abnormalities.

	Univariate Analysis		Multivariate Analysis	
	HR (95% CI)	*P*-value	HR (95% CI)	*P*-value
Age	1.04 (1.01–1.07)	0.003*	1.05 (1.01–1.09)	0.024*
Male gender	1.93 (0.73–5.10)	0.186	2.23 (0.57–8.72)	0.248
Hypertension	3.24 (1.12–8.90)	0.022*	0.98 (0.23–4.12)	0.980
Diabetes mellitus	1.99 (0.53–7.51)	0.312		
Chronic kidney disease	<0.1 (0.00->100)	1.00		
Coronary artery disease	3.50 (0.66–18.70)	0.143	0.58 (0.08–4.28)	0.590
Stroke	<0.1 (0.00->100)	1.00		
History of malignancy	0.00 (0.00->100)	1.000		
White cell count	1.04 (0.89–1.25)	0.722		
Lymphocyte count	1.18 (0.57–2.43)	0.655		
Alanine transaminase	0.98 (0.963–1.01)	0.118	0.98 (0.95–1.00)	0.069
Creatinine	1.02 (0.99–1.05)	0.134		

*p<0.05.

## Discussion

To the best of our knowledge, this study is the first systematic cardiovascular evaluation to detect subclinical and/or latent cardiac damage among COVID-19 survivors. Importantly, our study found that cardiac abnormalities were detected in 42.3% of the 97 COVID-19 survivors. The most common abnormality was sinus bradycardia with a smaller proportion of survivors with significant sinus bradycardia with heart rate below 50 bpm. However, the arrhythmia appeared to be self-limiting and gradually resolved over time. In the patients with serum troponin elevation, CMR did not show infarcts or clear evidences of myocarditis. In addition, newly detected atrial fibrillation, elevated NT-proBNP level, and impaired LVEF were detected in 1.0% of COVID-19 survivors. Lastly, non-specific T-wave abnormalities were newly detected in almost 10% of COVID-19 survivors for which the potential impact could be monitored. Our findings may help healthcare providers better understand the implications of COVID-19 when patients present for ongoing clinical care.

As we enter the deceleration phase of the pandemic with more patients recovering from the acute illness, a considerable proportion of COVID-19 patients reported to have cardiac damage during acute illness may imply a huge demand in subsequent cardiac care should these COVID-19 survivors develop heart failure or other cardiac complications in long term. There is an urgent need to understand the epidemiology of COVID-19 related cardiac sequela in order to define the need and scale of possible cardiac surveillance in COVID-19 survivors. In the investigation phase of the pandemic, myocardial injury as defined as an elevated troponin level was observed in 7% to 27.8% COVID-19 patients [2, 3, 7, 8, 19]. It was postulated to be due to acute myocarditis or injury secondary to an oxygen supply/demand mismatch particularly in those with pre-existing cardiovascular disease, i.e., type 2 myocardial infarction [20]. Nonetheless, many COVID-19 patients with biochemical evidence of myocardial injury did not have any overt cardiac manifestations and apparently recovered uneventfully. In concordance to previous studies, 10.1% of COVID-19 survivors in the present study had biochemical evidence of myocardial injury, and most had cardiovascular comorbidities, or even established cardiovascular diseases. Nonetheless, they did not have overt cardiac manifestations such as heart failure or acute coronary syndrome during COVID-19 hospitalization period. Interestingly, elevated troponin level above the 99^th^ percentile of upper reference limit remained detected in 6.2% COVID-19 survivors 1–4 weeks after hospital discharge. However, none had clinical heart failure, elevated NT-proBNP level or left ventricular systolic dysfunction detected during the screening. It is reassuring that despite the persistent troponin elevation, cardiac magnetic resonance imaging did not show infarction or myocarditis in these COVID-19 survivors. It has been shown that a significant proportion of COVID-19 patients with severe disease had lymphocytic myocarditis (14%) in autopsies [21], it is plausible that the persistent troponin elevation reflect on-going subclinical direct cytopathogenic and local pro-inflammatory effects of SARS-CoV-2 on the cardiomyocytes [6]. Notwithstanding, one (1.0%) COVID-19 survivor was detected to have impaired left ventricular systolic dysfunction with an elevated NT-proBNP level. While this is not surprising as similar correlation between influenza and heart failure has been well recognized [22], this highlights the need for vigilant monitoring and the importance of clinical suspicion for cardiovascular complications in COVID-19 survivors particularly in those with cardiovascular comorbidities.

The strikingly high prevalence of sinus bradycardia amongst COVID-19 survivors in the present study, contrasts with other respiratory viral infections such as influenza. Profound sinus bradycardia or sinus asystole leading to syncope have only been rarely reported in other viral infections including influenza [23], herpes simplex virus [24, 25], Ebola and West Nile virus [26, 27], attributed to acute myocarditis or autonomic nervous system involvement. In the outbreak of SARS due to SARS-CoV in 2003, studies from Canada and Hong Kong have also reported high prevalence of sinus bradycardia in hospitalized SARS patients as high as 27.5% [28, 29]. Significant sinus bradycardia with heart rate below 50 bpm were found in 14.9% SARS patients [29]. Although the mechanisms remain elusive, sinus bradycardia in SARS was transient and self-limiting, which resolves in 2–3 weeks [29]. Resembling SARS, sinus bradycardia in COVID-19 appears benign and recovers over a few weeks as found in the present study. More importantly, the absence of biochemical evidence of myocardial injury or heart failure during hospitalization and in cardiac surveillance clinic, and normal left ventricular function do not suggest any ongoing myocardial damage. A higher proportion of men than women among the COVID-19 survivors had sinus bradycardia. It has been shown that sex differences affect COVID-19 outcome, for instance male sex was associated with higher risk of death and intensive care unit admission [30]. Sex differences in the innate and adaptive immune system have been proposed [30], nonetheless it remains elusive how COVID-19 infection caused differential cardiac manifestation in different sexes.

Lastly, non-specific T-wave and ST segment abnormality was observed in 63.6% of COVID-19 patients with biochemical evidences of myocardial injury in the early case series [31]. T-wave inversion is associated with acute myocardial injury and underlying heart disease but isolated T-wave inversion in asymptomatic individuals does not appear to confer significant risk of adverse outcome or mortality. Amidst the pandemic, detail cardiac evaluation is often challenging, thereby the actual significance of these changes remain uncertain [31]. In the present study, 8.2% of COVID-19 survivors had newly detected T-wave inversions over the anterior leads. These changes was found in 1–3% adolescents particularly in female [32, 33], but only in 0.5% of middle-aged people [34]. Reassuringly, none of these COVID-19 survivors with T wave inversion had biochemical evidence of myocardial injury, elevated NT-proBNP level, or left ventricular dysfunction, despite the underlying pathophysiological mechanism remains elusive.

### Limitations

There are several limitations in our study. First, the presented data are collected from COVID-19 survivors without overt cardiac manifestation, therefore limiting the generalizability of the results to those patients with severe disease. Nonetheless, those with cardiac complications during the disease course do not need screening but aggressive guideline recommended treatment. Second, not all serum troponin levels during COVID-19 could be obtained, however we were able to describe these for approximately two-thirds of COIVD-19 survivors during the acute phase. Furthermore, while other inflammatory markers including C-reactive protein, ferritin, interleukin, interferon, etc., may allow better understanding of the underlying pathophysiological processes in COVID-19 related cardiac abnormalities, serum troponin and NT-proBNP concentration may remain the most widely available cardiac biomarkers for screening purposes. At the same time, standard 2D echocardiographic parameters were used to screen for possible myocardial damage in the current study, which is not as sensitive as the more advanced tissue doppler imaging derived global longitudinal strain. Third, as many patients did not have cardiac assessment prior to the index COVID-19 hospitalization, we could not ascertain the chronicity of these newly detected cardiac abnormalities. Fourth, the heterogeneity of time from hospital discharge to the cardiovascular screening may likewise affect the incidence and severity of newly detected cardiac abnormalities. Last but not least, there was not a control group of healthy subjects. Nonetheless, identification of these at-risk patients by systematic screening is the first critical step that would allow further prospective assessments to determine whether early intervention is associated with improved clinical outcomes. Further research is needed to assess the long-term benefits, harms, and value of expanded cardiac surveillance, use of surrogate cardiac biomarkers, and prophylactic cardioprotective therapy in COVID-19 survivors.

## Conclusion

This is the first systematic cardiac screening in COVID-19 survivors. It showed that cardiac abnormality is common amongst non-severe COVID-19 survivors without overt cardiac manifestations. Our findings may help healthcare providers better understand the cardiac implications of COVID-19 and define the necessity of cardiac surveillance amongst asymptomatic COVID-19 survivors.

## Supporting information

S1 File Deidentified clinical data of uncomplicated COVID-19 survivors. (SAV)

## Abbreviation list

CMR: Cardiac magnetic resonance imaging
COVID-19: Coronavirus Disease 2019
ECG: Electrocardiography
LGE: Late gadolinium enhancement
LVEF: Left ventricular ejection fraction
NT-proBNP: N-terminal-B-type natriuretic peptide
SARS-CoV-2: Severe Acute Respiratory Syndrome Coronavirus 2
